# Organizing MHC Class II Presentation

**DOI:** 10.3389/fimmu.2014.00158

**Published:** 2014-04-10

**Authors:** David R. Fooksman

**Affiliations:** ^1^Department of Pathology, Albert Einstein College of Medicine, Bronx, NY, USA

**Keywords:** MHC class II, diffusion, clustering, antigen presentation, microscopy, fluorescence

## Abstract

Major histocompatibility complex (MHC) class II molecules are ligands for CD4^+^ T cells and are critical for initiating the adaptive immune response. This review is focused on what is currently known about MHC class II organization at the plasma membrane of antigen presenting cells and how this affects antigen presentation to T cells. The organization and diffusion of class II molecules have been measured by a variety of biochemical and microscopic techniques. Membrane lipids and other proteins have been implicated in MHC class II organization and function. However, when compared with the organization of MHC class I or TCR complexes, much less is known about MHC class II. Since clustering of T cell receptors occurs during activation, the organization of MHC molecules prior to recognition and during synapse formation may be critical for antigen presentation.

## Introduction

Major histocompatibility complexes (MHC) class II molecules are comprised of two membrane bound chains loaded with a degenerate short peptide that provide both stability to the complex and diversity of unique complexes. These peptides are derived from self-proteins or from foreign proteins from pathogens, microbiota, and other organisms. CD4^+^ T cells recognize the peptide-loaded complexes through their T cell receptors (TCRs), which are optimized for binding MHC class II (MHC II) molecules, but diversified to recognize a repertoire of peptides TCR triggering is the critical checkpoint that determines which T cells are selected for activation and mobilization for the adaptive immune response. *In vivo*, T cells typically encounter these complexes in secondary lymphoid organs on the surfaces of professional antigen presenting cells (APCs) such as dendritic cells (DCs), B cells, and macrophages, as well as some mesenchymal cells such as lymphatic endothelial cells and stromal nodal cells. These encounters with MHC II are continuously occurring as T cells and some APCs are in constant cell migration, plasma membranes brushing up against each other in a crowded cell environment. The presence of additional cues, including chemokines, inflammatory cytokines (1, 2), and adhesion molecules and co-stimulatory ligands on APCs, can alert and direct T cells to activated APCs. In contrast, the absence of these cues can retard full T cell activation and induce tolerance.

The aim of this review is to present a comprehensive overview of what is currently known about MHC II organization at the plasma membrane of APC and how this affects antigen presentation to T cells. During the last 20 years, TCR organization and the consequences of clustering upon activation have received considerable attention. Compared to MHC class I (MHC I) (3), much less is known about MHC II organization and how it may or may not impact recognition by CD4^+^ T cells. Based on the recent findings on TCR clustering and 2D kinetics, it is likely that any perturbation of MHC II surface organization will have an impact on the quality of T cell activation. Many factors may contribute to the organization of MHC II molecules. These include accessory proteins, protein–lipid interactions, and the interplay of the cytoskeleton with plasma membrane. In addition, there are dynamics of trafficking that can alter the structure temporally. How the techniques and methodologies employed may contribute to our understanding of MHC II surface organization will also be discussed.

## MHC Class II Mobility and Diffusion

**Figure 1:**
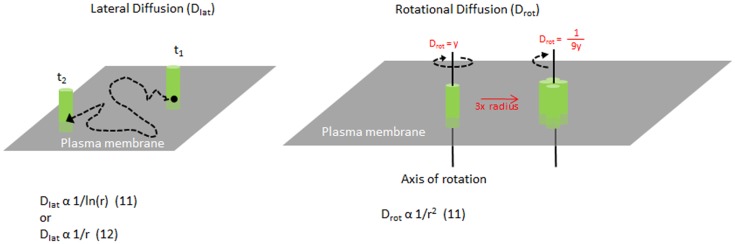
**Mobility of MHC molecules in the membrane**. Diagram illustrating lateral diffusion and rotational diffusion of membrane proteins such as MHC class II on the plasma membrane. Equations relating the size of the molecule and their diffusion rates are shown for each motion. For example, increasing the radius of a complex to 3× will change the *D*_rot_ by 1/9×.

As MHC II molecules are transmembrane proteins embedded within the plasma membrane, their movement is restricted by the lipid bilayer. MHC II molecules can move along the plane of the plasma membrane surface, known as lateral diffusion, or they can rotate around an axis perpendicular to the plasma membrane plane, known as rotational diffusion (Figure 1). These movements have speeds associated with them, referred to as diffusion coefficients, which are dependent on many factors intrinsic and extrinsic to the protein. In a living cell membrane, these include the size or oligomer status of the molecule or complex, the lipid environment and interactions with other proteins in and around the membrane, including the actin cytoskeleton. In general, larger complexes move more slowly, and interactions with other components typically impede mobility as well. By measuring the diffusion, one can investigate the organization of the molecules and the interactions that govern the protein mobility. Furthermore, MHC II mobility is likely to influence the efficiency of TCR binding (discussed later).

**Figure 2:**
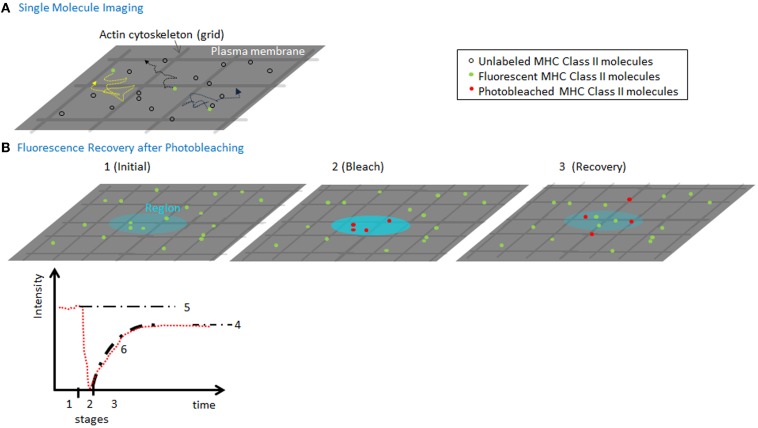
**Measuring MHC lateral diffusion**. **(A)** A diagram of single molecule imaging. A small fraction of molecules is labeled to ensure that only single molecules are visualized. High-speed cameras capture the position of the molecules, which can be used to track the speed and identify obstacles such as actin cytoskeleton forming “corrals.” By imaging at millisecond speeds can capture the tracks of molecules in between and during hops between actin corralled regions of the membrane. **(B)** A diagram of a FRAP experiment (1) using a low-power, attenuated laser, a region (~300 nm or larger) of the membrane is monitored for fluorescence of molecules using various detection instruments. (2) A pulse of non-attenuated high laser power is delivered to the spot, quickly photobleaching a population of molecules in the region. (3) The low-power monitoring is restored and the recovery of mobile fluorescent molecules to the region. The fluorescence intensity (in red) is plotted vs. time, to show the three time segments. Based on the final recovery (4) vs. the initial intensity (5), we can compute the mobile fraction. The fit of the recovery can be used to calculate a diffusion coefficient for lateral mobility. As the spot sizes are always larger than size actin meshwork, the rate of diffusion and recovery is sensitive to interactions with the cytoskeleton.

To determine MHC II lateral movement and organization at the plasma membrane, several quantitative and qualitative approaches have been employed including imaging. Fluorescence recovery after photobleaching (FRAP) is one commonly used technique, which can determine the fraction of molecules that are moving vs. bound/immobile molecules (mobile fraction) and the speed (*D*_lat_) of the mobile molecules (Figure 2). Both parameters can inform and are affected by the organization of the molecules, their association with other surface molecules, and engagement of cytoskeletal elements (4). This is true even for the smallest bleach spots (that are diffraction-limited) and provides an ensemble average speed for all molecules. In contrast, high-speed imaging of single molecules has been used to measure diffusion of individual MHC II complexes. Although these approaches are more technically challenging, the advantage is that one can measure micro diffusion, mobility at short timescales (tens of milliseconds), which can resolve the contributions of the actin meshwork on the movement of the molecules (5). This distinction can be useful if the cytoskeleton is inhibiting diffusion by corralling molecules, or by increasing the local concentration of molecules thereby stabilizing weak protein–protein interactions.

Several groups (6–9) have measured MHC class II mobility by single molecule tracking, which resolves dynamics at intervals in the tens of milliseconds. Based on measurements for the diffusion of MHC class II expressed on CHO cells, as GPI-anchored or transmembrane molecules, most MHC molecules appear to be highly mobile and diffusing at rates similar to GPI-anchored proteins. Typically, transmembrane proteins, which can engage proteins on both leaflets of the membrane, diffuse more slowly than GPI-anchored proteins. Because this is not observed for MHC II molecules, it suggests they do not have strong interactions with the actin cytoskeleton or other membrane proteins. However, FRAP measurements of MHC class II mobility on primary B cells and B cell lines showed considerably slower diffusion, with more immobile molecules on the plasma membrane [summarized nicely in Table 1 of Ref. (7)]. This may be the result of technical differences in methodology or perhaps B cells express additional components that can interact with class II molecules causing a reduced mobility. CHO cells are model cells often used for reductionist studies for technical reasons, but they may not reflect the physiological behavior of these proteins on professional APCs.

## MHC Self-Organization

It remains unclear if MHC class II molecules are found as monomers or associated in clusters on the plasma membrane. FRET measurements between MHC II surface molecules on B cell lines indicate clustering but these experiments were performed with intact antibodies which may induce formation of dimers, under non-saturating staining conditions (10). Superantigen staphylococcal enterotoxin A, which can activate T cells in an antigen-independent manner, has been shown to induce clusters of MHC II molecules (11). This suggests that the clustered state of MHC II may regulate the sensitivity of antigen presentation as we and others have reported for MHC I (3, 12).

Measuring the size of MHC II clusters in live cell membranes can be challenging. The size of MHC clusters can be approximated by the mobility of the complex. Originally, Saffman and Delbruck described lateral diffusion to be inversely related to ln (radius) of the molecule in a membrane (13) (Figure 1). This has been challenged by more recent studies (14, 15) and remains unclear. However, the rotational diffusion is inversely proportional to the radius squared (13). Typically, single chain proteins in the membrane rotate on the order of 10^−5^ s. As the radius of a molecule or complex increases, the speed of rotation will reduce greatly, making it highly sensitive to any change in clustering or complex formation. Changes in rotation will alter the rate of binding to other molecules (16).

Previously, we measured the rotation of MHC I clusters to determine the size of large cluster generated by engineered heavy chains with multiple dimerization domains in the C terminal tail (17). Using polarized FRAP, we were able to detect the rotation of large clusters of MHC I after cross-linking them in live cells. There are technical obstacles to making such measurements *in vivo*, in particular, having a rigid fluorophore that can report on the movement of the complex and not simply the movement of a dye conjugate or fluorescent protein attached by a flexible linker. We embedded the GFP motif within the C-terminus followed by crosslinkable domains that where rigid once bound by the crosslinker molecule. Anisotropy has been used to measure to other ligand–receptor complexes (18) and the oligomerization of the EGF receptor (19). In principle, measuring changes in anisotropy using single particle tracking can be applied to study of the binding or unbinding of MHC molecules by TCR on opposed membranes.

How might clusters of MHC II affect TCR recognition? TCR microclusters are important during early events of T cell activation and antigen recognition (20). Activated T cells have pre-clustered TCR on their cell surface, which enhance binding of peptide–MHC I complexes (21). Clustering of MHC II monomers would change their lateral diffusion and their rotation from tens of microseconds into the millisecond timescale, making them essentially rotationally immobile, in comparison to the TCRs on the opposing surface (Figure 1). Slower rotating clusters of ligands or receptors should have slower rates of association and dissociation (16), which in theory, could facilitate rebinding events and may alter the duration of TCR signaling.

## MHC–Protein Interactions

Upon maturation, DCs and B cells express higher surface levels of MHC I and II molecules, B7-family co-stimulatory molecules and adhesion molecules, which enhances antigen presentation. MHC class II molecules associate with various membrane proteins on the cell surface, including ICAM-1 and MHC I based on FRET measurements (22). Recruitment of ICAM-1 and ICAM-3 to the immunological synapse can also drive MHC class II accumulation, indicating a protein–protein interaction (23). Changes in MHC class II levels during maturation are the result of a reduced endocytosis (24) of molecules and a change in ubiquitination, which promotes plasma membrane tropism vs. endosome or multi-vesicular body localization (25). Trafficking of vesicles densely packed with MHC class II complexes, arrive at the plasma membrane as clusters (26), as is the case for class I molecules (27). These structures dissipate over time but can provide hotspots of nascent ligands for binding TCR.

## Actin

The role of actin in MHC mobility and presentation is complex. Typically, the cortical actin meshwork can provide barrier to membrane mobility or even immobilize certain proteins (5). Treatment with cytochalasin D did not affect the diffusion of the MHC class II molecules (6–9) in CHO cells. Similarly, inhibition of actin by cytochalasin D treatment did not affect T cell activation (28) using the CH27 B cell line. However, blockade of actin with latrunculin B in CH27 cells reduced synapse recruitment of PKC-theta on T cells (29). Treatment with latrunculin A also increased class II diffusion (7). These contrasting results may reflect differences in cell types used or differences in experimental design. For example, latrunculins depolymerize actin filaments, while cytochalasin D blocks actin polymerization.

## Lipid Domains and Other Microdomains

Like many other membrane ligands and receptors, there has been interest in how lipid rafts affect MHC class II organization. Raft lipids and raft-associated proteins are found in the detergent-insoluble fraction of a sucrose gradient in contrast to other membrane proteins and lipids that can distribute through the gradient as a function of their molecular weight or specific gravity. These experiments are notoriously controversial as they require specific detergents to separate the fractions, and these raft domains are not readily visible by imaging without the aid of fluorescent lipid probes that tend to aggregate and possibly nucleate these domain structures (30, 31). The lifetime and size of these structures in live membranes may be much smaller when unperturbed.

A proportion of MHC class II can be found in lipid rafts as measured by detergent extraction followed by sucrose gradient centrifugation (32, 33). The fraction of detergent-insoluble MHC II is reduced upon DC maturation (34). Disruption of membrane cholesterol or membrane structure with methyl-β-cyclodextrin or nystatin reduces MHC II association with raft lipids and correlates with reduced antigen presentation. Typically, cross-linking membrane receptors can induce the accumulation of these molecules into the detergent resistant fraction associated with lipid rafts. Lipid composition affects MHC presentation, through cholesterol binding (35, 36). We do not know if APC membrane composition directly affects T cells, or whether the changes are indirectly contributing to MHC mobility, protein–protein interactions, or other biological parameters (31). Nevertheless, it is clear that certain membrane proteins are sensitive to the lipid environment and disruptions in lipid organization affect T cell recognition.

Various tetraspanins have also been implicated in MHC class II processing in endosomes and presentation. Within endosomal compartments, CD9, CD63, and CD81 all were important for controlling surface expression of MHC II (37). Tetraspanins can also associate with class II molecules on the surface (38). Vogt and colleagues (39) have reported that about 10% of class II molecules are found in “CDw78 microdomains” that are not lipid raft associated, and can be stained with the antibody CDw78. Although the exact determinant or epitope of the antibody is unclear, it seems to recognize multimeric class II molecules or complexes with tetraspanins. Cross-linking MHC II and CD48 on the surface (29) can recruit glycosphingolipid-rich domains and induce the accumulation of f-actin and phosphotyrosine at the plasma membrane of the APC.

## MHC Molecules on Dendritic Cells

There is a dearth of measurements of MHC class II diffusion and the role of actin on DCs, which are the most potent APCs in the body. As DC mature, many changes in cell morphology are induced including increased membrane protrusions and ruffling and increased polymerized actin (40) leading to more dendrite activity and better presentation. These morphological changes are most likely aimed at enhancing antigen presentation and engagement with T cells, but this is only speculation. In one study, knock down of gelsolin, an actin regulator protein was found to have no effect on antigen presentation in DCs (41) based on *in vitro* assays of antigen uptake and presentation. It is unknown if DC maturation alters MHC class II mobility and organization while upregulating ligand numbers on the surface. Moreover, it may be interesting to see whether MHC class II mobility varies on DC subpopulations, which have different potentials for activating or tolerizing T cells.

Dendritic cells present antigens to highly motile antigen-specific T cells in a dynamic environment. *In vivo*, the dosage and potency of peptide–MHC complexes on DCs can mediate T cell arrest or continued scanning, thus influencing the balance between tolerance and activation (42–45). In these complex environments, the organization of MHC molecules can tip the balance between T cell recognition or neglect.

## Relationship between TCR Triggering and MHC Organization

T cell triggering has been of great interest for several decades with too many seminal findings to discuss here. With the improvements in high resolution imaging, artificial APCs based on planar bilayer substrates or engineered cell lines, recent studies have been able to better determine biophysical and molecular components of T cell activation. These discoveries on the T cell side may suggest how the structure and organization of MHC molecules might influence TCR recognition.

In the past decade, we have determined that TCR microclusters are formed when agonist peptide–MHC complexes are presented to T cells (46). These microclusters are the signaling quanta for the T cell, comprised of multiple TCRs, associated with co-receptors CD4 or CD8, signaling kinases such as Lck and Zap-70, with some complexes engaging adaptor molecules such as SLP-76 and LAT, leading to the activation of signaling cascades, fluxing of calcium, and ultimately gene expression. TCR microclusters are maintained and trafficked along the plasma membrane through actin polymerization and contractions of actin by myosin IIa until they are internalized and degraded in multi-vesicular body pathways. From formation until degradation, these microclusters generate active signaling mediated by the kinases and adaptors associated with them. Sustained signaling is dependent on the continued formation of microclusters and the lifetime of the microcluster and factors that impede either reduce total signaling. For example, blocking MHC ligands with antibody after initial formation of microclusters inhibits the generation of new clusters (20). Interfering with the actin cytoskeleton with latrunculin A disperses clusters and terminates signaling. In contrast, blocking internalization and degradation of microclusters, such as by interfering with Tsg101 mRNA, enhances T cell signaling (47). Based on these studies, we can postulate that factors controlling the distribution and diffusion of MHC and co-stimulatory molecules on professional APCs could have consequences on the lifetime or formation of TCR microclusters. Cytoskeleton engagement or diffusion barriers on the APCs could block MHC escape from TCR microclusters, thereby enhancing T cell signaling.

There has been considerable interest in understanding the basis of how MHC recognition induces TCR triggering. It is well known that soluble antibody against CD3 subunits can induce T cell activation, either by clustering receptors or inducing some mechanical force. However, at longer distances between antibody Fab fragments, TCR triggering does not occur (48). A recent study (49) investigated the stoichiometry of MHC and TCR interactions by using lipid-tethered MHCs on planar bilayers with defined concentration that were confined to grids patterns. From these experiments they determined that a minimum of two agonist peptide–MHC complexes are required per grid, to be in contact with a TCR microcluster and trigger calcium signaling. Vale and colleagues using TCR zeta chain fused to a mutated FK506-binding domain, or FKBP, could induce T cell signaling via Zap-70 in the absence of MHC binding, antibody cross-linking or any other extracellular stimulus simply by clustering the receptor (50) via FKBP dimerization. These two results indicate that TCR triggering may simply require clustering and that two TCR units with their associated CD3 chains may be the minimal structure required for activation. Based upon these studies, a clustered presentation of MHC molecules could potentially enhance TCR signaling by recruiting additional TCRs into a complex.

In contrast, work focused on understanding the biophysical parameters of MHC–TCR binding that correlate with triggering has questioned long standing paradigms previously held. Initial measurements of MHC–TCR binding, have correlated slower off-rates of binding (or long half-life of interaction) with better T cell activation (51), with antagonists, in general, having intermediate off-rates compared to agonists and null peptides (52). These measurements were conducted in solution in 3D, with all forms of motion for both molecules in play. In recent years, focus has shifted to trying to understand binding with motion limited to lateral and rotation diffusion in the plasma membranes. Studies (53, 54) using various optical and mechanical assays to study these interactions have demonstrated that MHC and TCR binding behaves quite different under these conditions. TCR–MHC on-rates are faster than in 3D solution, as these molecules transit in parallel sheets. MHC molecules with agonist peptide have off-rates comparable to weaker agonist ligands but even faster on-rates, driving activation. These measurements have implications with regard to MHC mobility and cluster formation. If fast binding is critical, clusters of MHC may actually inhibit the efficiency of recognition, particularly clusters containing various peptides.

## Concluding Remarks

Further investigation is required to understand the organization of MHC class II molecules, particularly on real APCs. Compared to MHC I or TCR organization, much less is known about class II organization and how it may impact antigen presentation. Based on the recent findings on TCR clustering and 2D kinetics, it is likely that any perturbation of MHC class II surface organization will have an impact on T cell recognition, but will it enhance or inhibit? Do DCs regulate MHC class II organization in responses to maturation or inflammation? Can pathogens target organization to modify antigen presentation as they seem to do for MHC class I molecules (12)? By modulating MHC II organization, we should be able to better control T cell activation to generate more effective anti-tumor responses and long-lived immunological memory in the future.

## Conflict of Interest Statement

The Guest Associate Editor Laura Santambrogio declares that, despite being affiliated to the same institution as author David R. Fooksman, the review process was handled objectively and no conflict of interest exists. The author declares that the research was conducted in the absence of any commercial or financial relationships that could be construed as a potential conflict of interest.

## References

[1] Castellino F Huang AY Altan-Bonnet G Stoll S Scheinecker C Germain RN Chemokines enhance immunity by guiding naive CD8+ T cells to sites of CD4+ T cell-dendritic cell interaction. Nature (2006) 440:890–5 10.1038/nature04651 16612374

[2] Kastenmuller W Brandes M Wang Z Herz J Egen JG Germain RN Peripheral prepositioning and local CXCL9 chemokine-mediated guidance orchestrate rapid memory CD8+ T cell responses in the lymph node. Immunity (2013) 38:502–13 10.1016/j.immuni.2012.11.012 23352234 PMC3793246

[3] Fooksman DR Gronvall GK Tang Q Edidin M Clustering class I MHC modulates sensitivity of T cell recognition. J Immunol (2006) 176:6673–80 16709826 10.4049/jimmunol.176.11.6673 PMC1524854

[4] Lenne PF Wawrezinieck L Conchonaud F Wurtz O Boned A Guo XJ Dynamic molecular confinement in the plasma membrane by microdomains and the cytoskeleton meshwork. EMBO J (2006) 25:3245–56 10.1038/sj.emboj.7601214 16858413 PMC1523176

[5] Kusumi A Nakada C Ritchie K Murase K Suzuki K Murakoshi H Paradigm shift of the plasma membrane concept from the two-dimensional continuum fluid to the partitioned fluid: high-speed single-molecule tracking of membrane molecules. Annu Rev Biophys Biomol Struct (2005) 34:351–78 10.1146/annurev.biophys.34.040204.144637 15869394

[6] Nishimura SY Vrljic M Klein LO McConnell HM Moerner WE Cholesterol depletion induces solid-like regions in the plasma membrane. Biophys J (2006) 90:927–38 10.1529/biophysj.105.070524 16272447 PMC1367117

[7] Umemura YM Vrljic M Nishimura SY Fujiwara TK Suzuki KG Kusumi A Both MHC class II and its GPI-anchored form undergo hop diffusion as observed by single-molecule tracking. Biophys J (2008) 95:435–50 10.1529/biophysj.107.123018 18339737 PMC2426619

[8] Vrljic M Nishimura SY Brasselet S Moerner WE McConnell HM Translational diffusion of individual class II MHC membrane proteins in cells. Biophys J (2002) 83:2681–92 10.1016/S0006-3495(02)75277-6 12414700 PMC1302352

[9] Vrljic M Nishimura SY Moerner WE McConnell HM Cholesterol depletion suppresses the translational diffusion of class II major histocompatibility complex proteins in the plasma membrane. Biophys J (2005) 88:334–47 10.1529/biophysj.104.045989 15516525 PMC1305010

[10] Gombos I Detre C Vamosi G Matko J Rafting MHC-II domains in the APC (presynaptic) plasma membrane and the thresholds for T-cell activation and immunological synapse formation. Immunol Lett (2004) 92:117–24 10.1016/j.imlet.2003.11.022 15081535

[11] Narayan K Perkins EM Murphy GE Dalai SK Edidin M Subramaniam S Staphylococcal enterotoxin A induces small clusters of HLA-DR1 on B cells. PLoS One (2009) 4:e6188 10.1371/journal.pone.0006188 19587800 PMC2705189

[12] Ferez M Castro M Alarcon B van Santen HM Cognate peptide-MHC complexes are expressed as tightly apposed nanoclusters in virus-infected cells to allow TCR crosslinking. J Immunol (2014) 192:52–8 10.4049/jimmunol.1301224 24307729

[13] Saffman PG Delbruck M Brownian motion in biological membranes. Proc Natl Acad Sci U S A (1975) 72:3111–3 10.1073/pnas.72.8.3111 1059096 PMC432930

[14] Gambin Y Lopez-Esparza R Reffay M Sierecki E Gov NS Genest M Lateral mobility of proteins in liquid membranes revisited. Proc Natl Acad Sci U S A (2006) 103:2098–102 10.1073/pnas.0511026103 16461891 PMC1413751

[15] Guigas G Weiss M Size-dependent diffusion of membrane inclusions. Biophys J (2006) 91:2393–8 10.1529/biophysj.106.087031 16829562 PMC1562383

[16] Hill TL Effect of rotation on the diffusion-controlled rate of ligand-protein association. Proc Natl Acad Sci U S A (1975) 72:4918–22 10.1073/pnas.72.12.4918 1061081 PMC388844

[17] Fooksman DR Edidin M Barisas BG Measuring rotational diffusion of MHC class I on live cells by polarized FPR. Biophys Chem (2007) 130:10–6 10.1016/j.bpc.2007.06.013 17656002 PMC2094112

[18] de Jong LA Uges DR Franke JP Bischoff R Receptor-ligand binding assays: technologies and applications. J Chromatogr B Analyt Technol Biomed Life Sci (2005) 829:1–25 10.1016/j.jchromb.2005.10.002 16253574

[19] de Heus C Kagie N Heukers R van Bergen en Henegouwen PM Gerritsen HC Analysis of EGF receptor oligomerization by homo-FRET. Methods Cell Biol (2013) 117:305–21 10.1016/B978-0-12-408143-7.00016-5 24143984

[20] Varma R Campi G Yokosuka T Saito T Dustin ML T cell receptor-proximal signals are sustained in peripheral microclusters and terminated in the central supramolecular activation cluster. Immunity (2006) 25:117–27 10.1016/j.immuni.2006.04.010 16860761 PMC1626533

[21] Fahmy TM Bieler JG Edidin M Schneck JP Increased TCR avidity after T cell activation: a mechanism for sensing low-density antigen. Immunity (2001) 14:135–43 10.1016/S1074-7613(01)00096-6 11239446

[22] Bene L Balazs M Matko J Most J Dierich MP Szollosi J Lateral organization of the ICAM-1 molecule at the surface of human lymphoblasts: a possible model for its co-distribution with the IL-2 receptor, class I and class II HLA molecules. Eur J Immunol (1994) 24:2115–23 10.1002/eji.1830240928 7916294

[23] de la Fuente H Mittelbrunn M Sanchez-Martin L Vicente-Manzanares M Lamana A Pardi R Synaptic clusters of MHC class II molecules induced on DCs by adhesion molecule-mediated initial T-cell scanning. Mol Biol Cell (2005) 16:3314–22 10.1091/mbc.E05-01-0005 15872088 PMC1165413

[24] Wilson NS El-Sukkari D Villadangos JA Dendritic cells constitutively present self antigens in their immature state in vivo and regulate antigen presentation by controlling the rates of MHC class II synthesis and endocytosis. Blood (2004) 103:2187–95 10.1182/blood-2003-08-2729 14604956

[25] van Niel G Wubbolts R Ten Broeke T Buschow SI Ossendorp FA Melief CJ Dendritic cells regulate exposure of MHC class II at their plasma membrane by oligoubiquitination. Immunity (2006) 25:885–94 10.1016/j.immuni.2006.11.001 17174123

[26] Bosch B Heipertz EL Drake JR Roche PA Major histocompatibility complex (MHC) class II-peptide complexes arrive at the plasma membrane in cholesterol-rich microclusters. J Biol Chem (2013) 288:13236–42 10.1074/jbc.M112.442640 23532855 PMC3650363

[27] Gheber LA Edidin M A model for membrane patchiness: lateral diffusion in the presence of barriers and vesicle traffic. Biophys J (1999) 77:3163–75 10.1016/S0006-3495(99)77147-X 10585938 PMC1300587

[28] Wulfing C Sjaastad MD Davis MM Visualizing the dynamics of T cell activation: intracellular adhesion molecule 1 migrates rapidly to the T cell/B cell interface and acts to sustain calcium levels. Proc Natl Acad Sci U S A (1998) 95:6302–7 10.1073/pnas.95.11.6302 9600960 PMC27665

[29] Gordy C Mishra S Rodgers W Visualization of antigen presentation by actin-mediated targeting of glycolipid-enriched membrane domains to the immune synapse of B cell APCs. J Immunol (2004) 172:2030–8 14764667 10.4049/jimmunol.172.4.2030

[30] Edidin M The state of lipid rafts: from model membranes to cells. Annu Rev Biophys Biomol Struct (2003) 32:257–83 10.1146/annurev.biophys.32.110601.142439 12543707

[31] Huby R Chowdhury F Lombardi G Rafts for antigen presentation? Nat Immunol (2001) 2:3 10.1038/83154 11135565

[32] Anderson HA Hiltbold EM Roche PA Concentration of MHC class II molecules in lipid rafts facilitates antigen presentation. Nat Immunol (2000) 1:156–62 10.1038/77842 11248809

[33] Khandelwal S Roche PA Distinct MHC class II molecules are associated on the dendritic cell surface in cholesterol-dependent membrane microdomains. J Biol Chem (2010) 285:35303–10 10.1074/jbc.M110.147793 20833718 PMC2975154

[34] Setterblad N Roucard C Bocaccio C Abastado JP Charron D Mooney N Composition of MHC class II-enriched lipid microdomains is modified during maturation of primary dendritic cells. J Leukoc Biol (2003) 74:40–8 10.1189/jlb.0103045 12832441

[35] Roy K Ghosh M Pal TK Chakrabarti S Roy S Cholesterol lowering drug may influence cellular immune response by altering MHC II function. J Lipid Res (2013) 54:3106–15 10.1194/jlr.M041954 24038316 PMC3793615

[36] Rockett BD Melton M Harris M Bridges LC Shaikh SR Fish oil disrupts MHC class II lateral organization on the B-cell side of the immunological synapse independent of B-T cell adhesion. J Nutr Biochem (2013) 24:1810–6 10.1016/j.jnutbio.2013.02.013 23791516 PMC3785547

[37] Hoorn T Paul P Janssen L Janssen H Neefjes J Dynamics within tetraspanin pairs affect MHC class II expression. J Cell Sci (2012) 125:328–39 10.1242/jcs.088047 22302999

[38] Szollosi J Horejsi V Bene L Angelisova P Damjanovich S Supramolecular complexes of MHC class I, MHC class II, CD20, and tetraspan molecules (CD53, CD81, and CD82) at the surface of a B cell line JY. J Immunol (1996) 157:2939–46 8816400

[39] Kropshofer H Spindeldreher S Rohn TA Platania N Grygar C Daniel N Tetraspan microdomains distinct from lipid rafts enrich select peptide-MHC class II complexes. Nat Immunol (2002) 3:61–8 10.1038/ni750 11743588

[40] Shutt DC Daniels KJ Carolan EJ Hill AC Soll DR Changes in the motility, morphology, and F-actin architecture of human dendritic cells in an in vitro model of dendritic cell development. Cell Motil Cytoskeleton (2000) 46:200–21 10.1002/1097-0169(200007)46:3<200::AID-CM5>3.0.CO;2-M 10913967

[41] West MA Antoniou AN Prescott AR Azuma T Kwiatkowski DJ Watts C Membrane ruffling, macropinocytosis and antigen presentation in the absence of gelsolin in murine dendritic cells. Eur J Immunol (1999) 29:3450–5 10.1002/(SICI)1521-4141(199911)29:11<3450::AID-IMMU3450>3.0.CO;2-A 10556799

[42] Skokos D Shakhar G Varma R Waite JC Cameron TO Lindquist RL Peptide-MHC potency governs dynamic interactions between T cells and dendritic cells in lymph nodes. Nat Immunol (2007) 8:835–44 10.1038/ni1490 17632517

[43] Shakhar G Lindquist RL Skokos D Dudziak D Huang JH Nussenzweig MC Stable T cell-dendritic cell interactions precede the development of both tolerance and immunity in vivo. Nat Immunol (2005) 6:707–14 10.1038/ni1210 15924144 PMC1560107

[44] Henrickson SE Perro M Loughhead SM Senman B Stutte S Quigley M Antigen availability determines CD8(+) T cell-dendritic cell interaction kinetics and memory fate decisions. Immunity (2013) 39:496–507 10.1016/j.immuni.2013.08.034 24054328 PMC3914670

[45] Marangoni F Murooka TT Manzo T Kim EY Carrizosa E Elpek NM The transcription factor NFAT exhibits signal memory during serial T cell interactions with antigen-presenting cells. Immunity (2013) 38:237–49 10.1016/j.immuni.2012.09.012 23313588 PMC3582823

[46] Fooksman DR Vardhana S Vasiliver-Shamis G Liese J Blair DA Waite J Functional anatomy of T cell activation and synapse formation. Annu Rev Immunol (2010) 28:79–105 10.1146/annurev-immunol-030409-101308 19968559 PMC2885351

[47] Vardhana S Choudhuri K Varma R Dustin ML Essential role of ubiquitin and TSG101 protein in formation and function of the central supramolecular activation cluster. Immunity (2010) 32:531–40 10.1016/j.immuni.2010.04.005 20399684 PMC2905630

[48] Cochran JR Cameron TO Stern LJ The relationship of MHC-peptide binding and T cell activation probed using chemically defined MHC class II oligomers. Immunity (2000) 12:241–50 10.1016/S1074-7613(00)80177-6 10755611

[49] Manz BN Jackson BL Petit RS Dustin ML Groves J T-cell triggering thresholds are modulated by the number of antigen within individual T-cell receptor clusters. Proc Natl Acad Sci U S A (2011) 108:9089–94 10.1073/pnas.1018771108 21576490 PMC3107331

[50] James JR Vale RD Biophysical mechanism of T-cell receptor triggering in a reconstituted system. Nature (2012) 487:64–9 10.1038/nature11220 22763440 PMC3393772

[51] Krummel M Wulfing C Sumen C Davis MM Thirty-six views of T-cell recognition. Philos Trans R Soc Lond B Biol Sci (2000) 355:1071–6 10.1098/rstb.2000.0644 11186308 PMC1692810

[52] Lyons DS Lieberman SA Hampl J Boniface JJ Chien Y Berg LJ A TCR binds to antagonist ligands with lower affinities and faster dissociation rates than to agonists. Immunity (1996) 5:53–61 10.1016/S1074-7613(00)80309-X 8758894

[53] Huang J Zarnitsyna VI Liu B Edwards LJ Jiang N Evavold BD The kinetics of two-dimensional TCR and pMHC interactions determine T-cell responsiveness. Nature (2010) 464:932–6 10.1038/nature08944 20357766 PMC2925443

[54] Huppa JB Axmann M Mortelmaier MA Lillemeier BF Newell EW Brameshuber M TCR-peptide-MHC interactions in situ show accelerated kinetics and increased affinity. Nature (2010) 463:963–7 10.1038/nature08746 20164930 PMC3273423

